# The relationship between self-stigma and sociodemographic variables in people with substance abuse

**DOI:** 10.1186/1940-0640-7-S1-A43

**Published:** 2012-10-09

**Authors:** Pollyanna Silveira, Gabriela Ferreira, Rhaisa Soares, Flaviane Felicissimo, Fabricia Nery, Ana L Casela, Érika Monteiro, Telmo Ronzani, Ana R Noto

**Affiliations:** 1 School of Medicine, Federal University of São Paulo, São Paulo, Brazil; 2 Department of Social Psychology and Public Health, Federal University of Juiz de Fora, Juiz de Fora, Brazil; 3 Department of Psychology, Federal University of Juiz de Fora, Juiz de Fora, Brazil; 4 Departament of Psychobiology, Paulista School of Medicine, São Paulo, Brazil

## 

Self-stigma occurs when members of a stigmatized group internalize negative views of themselves, resulting in devaluation and withdrawal. Studies have shown that people with substance use are heavily stigmatized and may internalize these negative beliefs about themselves. This study aimed to evaluate the relationship between self-stigma and sociodemographic variables such as age, years of education, religious practice, and employment status. The instruments used were the Internalized Stigma of Mental Illness (ISMI) scale adapted for substance users and a sociodemographic questionnaire developed by the authors, both applied in face-to-face interviews. The sample was composed by 248 patients diagnosed with substance abuse participating in treatment in Juiz de Fora, Brazil. Ninety-four percent of participants were male with a mean age of 35.3 years (SD = 10.9); 69.1% were unemployed, with the mean duration of their education of 7.9 years (SD = 4.8), and 51.3% were not practicing any religion. Associations between sociodemographic variables and internalized stigma were examined using the Spearman correlation test and t-test for independent samples. For all the analyses, we assumed confidence intervals of 95% (p < 0.05). The ISMI scores were not correlated to any of the sociodemographic variables, suggesting that certain psychosocial variables have greater influence on the internalization of stigma. However, analyses revealed that the only variable showing a difference between the means was employment status (t = -2.06; p < 0.05), indicating that people who are unemployed have greater self-stigma than people who are working. This may be due to employed persons having a greater inclusion in a social environment, which promotes a higher quality of social interactions. These results highlight the need to identify variables influencing self-stigma among people with drug use in order to develop interventions that lessen self-stigma more effectively.

